# Supplementary material for: Identification of photoperiod-induced specific miRNAs in the adrenal glands of Sunite sheep (Ovis aries)
Source: Front Vet Sci. 2022 Jul 22;9:888207. doi: 10.3389/fvets.2022.888207 (PMC9354845; doi:10.3389/fvets.2022.888207)
Supplement: Supplementary file 1 [file Data_Sheet_1.ZIP › Supplementary file/miRDeep2 file.zip!/miRDeep2 file/Know_Structure/SP42A1/oar-mir-668.pdf]

miRBase precursor : oar-mir-668
Total read count : 17
oar-miR-668-5p read count : 3
oar-miR-668-3p read count : 14
remaining reads : 0

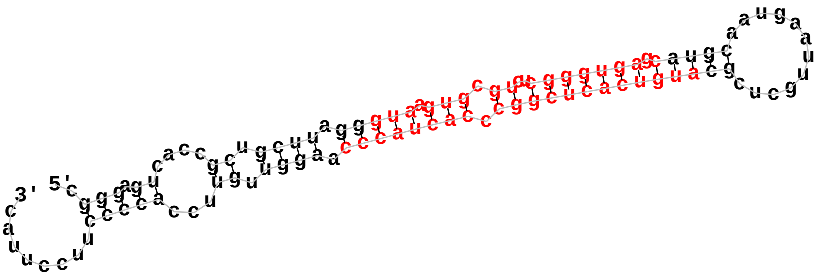

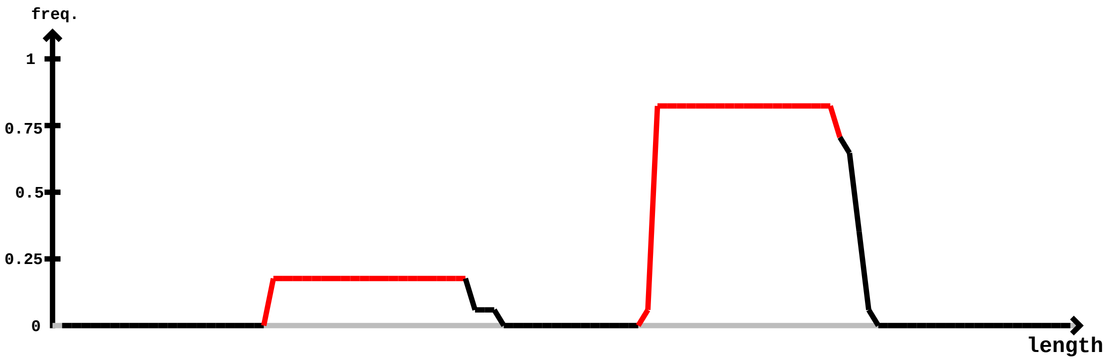


```
                                                              oar-miR-668-3p
                    oar-miR-668-5p
5'- cgggagucaccgcugcuuaggguaagugcgucucgggugagcaugcaaugaauugcucgcaugucacucggcccacuacccaagguuguuccaccccuuccuuac   -3'    exp
    .(((.((....((.((((.(((((.(((.((..(((((((.(((((............)))))))))))))).)))))))).)))).))...))))).......   reads      mm         sample
    .....................guaagugcgucucgggugagc...............................................................   2          0          seq
    .....................guaagugcgucucgggugagcaug............................................................   1          0          seq
    ............................................................augucacucggcccacuaccc........................   1          0          seq
    .............................................................ugucacucggcccacuacc.........................   2          0          seq
    .............................................................ugucacucggcccacuaccca........................   5          0          seq
    .............................................................ugucacucggcccacuacccaa.......................   5          0          seq
    .............................................................ugucacucggcccacuacccaag.....................   1          0          seq
```
